# Uptake and tissue accretion of orally administered free carboxylic acid as compared to ethyl ester form of docosahexaenoic acid (DHA) in the rat

**DOI:** 10.1371/journal.pone.0201367

**Published:** 2018-08-02

**Authors:** Anna Lindblom, Cecilia Ericsson, Therese Hagstedt, Ann Kjellstedt, Jan Oscarsson, Nicholas D. Oakes

**Affiliations:** 1 Innovative Medicines Early Development, Cardiovascular Renal & Metabolism, Bioscience, AstraZeneca R&D Gothenburg, Mölndal, Sweden; 2 Early Clinical Development, AstraZeneca R&D Gothenburg, Mölndal, Sweden; 3 Global Medicines Early Development, Cardiovascular Renal & Metabolism, Metabolism, AstraZeneca R&D Gothenburg, Mölndal, Sweden; Universite du Quebec a Montreal, CANADA

## Abstract

**Aim:**

The aim of this study was to compare the plasma exposure and tissue accretion of docosahexaenoic acid (DHA) in response to oral dosing of free carboxylic acid (OM3CA) and ethyl ester (OM3EE) forms.

**Materials and methods:**

Sixteen adult male Wistar rats, fed a low-fat, carbohydrate-rich, standard chow diet, were chronically catheterized and gavaged for 5 consecutive days with either OM3CA (n = 9) or OM3EE (n = 7), the last day fasted overnight and spiked respectively with either ^14^C-DHA or ^14^C-DHA-ethyl ester (^14^C-DHA-EE) tracers. Appearance of ^14^C-labelled plasma polar and neutral lipids over 4 h and retention of ^14^C-activity (*R*) in the tissues at 4 h were measured.

**Results:**

Compared to OM3EE, OM3CA resulted in 2- and 3-fold higher areas under the plasma ^14^C-labelled polar and neutral lipid curves (exposures), respectively, as well as, higher *R* in all tissues examined. For both OM3CA and OM3EE, *R* varied in a tissue specific manner; highest in liver, followed by red skeletal muscle, adipose tissue, brain and white skeletal muscle. Multiple linear regression analysis revealed that *R* in each tissue (except liver) was dependent on polar lipid exposure alone (r^2^>0.87 and P<0.001), but not neutral lipid exposure, and furthermore this dependence was indistinguishable for OM3CA and OM3EE. In the liver, *R* was found to be dependent on both polar and neutral lipid exposures (r^2^ = 0.97, P<0.001), with relative contributions of 85±2% and 15±2%, respectively. As for the other tissues, these dependencies were indistinguishable for OM3CA and OM3EE.

**Conclusion:**

The present results, in fasted low-fat diet fed rats, are consistent with higher oral bioavailability of OM3CA versus OM3EE forms of DHA. Once DHA has entered the circulation, the tissue distribution is independent of the dosed form and uptake in the skeletal muscle, fat and brain is driven by the polar pools of DHA in plasma, while DHA accretion in liver is supplied by both polar and neutral plasma lipids.

## Introduction

Since the initial finding of reduced platelet aggregation by fish oil [1], numerous studies have shown that oral supplementation with omega-3 fatty acids have positive effects on plasma lipoproteins [2], inflammatory diseases [3], and potentially also positive effects on cardiovascular disease [2, 4] and neurological disorders [5].

The main omega-3 fatty acids in prescription grade formulations are docosahexaenoic acid (DHA) and eicosapentaenoic acid (EPA). The intestinal digestion, absorption and uptake of these n-3 polyunsaturated fatty acids depend on the chemical form of the fatty acids administered, including triglycerides, phospholipids, ethyl esters or free fatty acids (FFA) as shown in clinical [6–11] and animal studies [12–14]. The difference in uptake between the different chemical forms is to some extent explained by differences in hydrolysis of the ester bonds by pancreatic lipase; ethyl esters being hydrolyzed more slowly compared to other chemical forms of omega-3 fatty acids by pancreatic lipase [11]. Moreover, not only intestinal absorption, but also the metabolic fate may be dependent on the chemical form of the fatty acids as exemplified by greater tissue deposition and lower β-oxidation of DHA in rats given DHA esterified in phospholipids as compared to triglycerides despite similar bioavailability [15]. Similar results were obtained in another study using radiolabeled DHA esters; oral 2-DHA-phosphatidylcholine resulted in a higher accretion of DHA in the liver than 2-DHA-triglyceride [16]. In summary, not only differences in bioavailability, but also differences in absorption rate and re-esterification of different omega-3 fatty acid esters might influence tissue accretion. Differences in tissue accretion are of potential importance because if such differences occur in key target tissues (e.g. liver or brain), it may result in different therapeutic efficacy.

In a rat study, plasma DHA exposure was higher following dosing of the FFA form as compared with the ethyl ester form [12]. In humans, systemic bioavailability of free omega-3 carboxylic acids (OM3CA) was higher than omega-3 fatty acid ethyl esters (OM3EE), as studied in the fasted state after a period of low-fat diet [9, 10]. However, to the best of our knowledge, no study has investigated both bioavailability and tissue accretion comparing free DHA and DHA ethyl esters.

The aim in this study was to compare bioavailability and tissue distribution of free DHA and the ethyl ester forms of DHA (DHA-EE). In our study, clinically applied formulations of omega-3 fatty acids, based on mixtures of DHA and EPA, were used as sources of dosing material for the OM3CA and OM3EE groups. While the groups received almost equimolar doses of the total of both DHA and EPA, the relative contributions of DHA and EPA in the groups differed. Rats were given a low fat diet and orally dosed with either OM3EE or OM3CA for 5 days. After an overnight fast to standardize the measurements of uptake and tissue accretion of DHA, plasma exposure and tissue accretion of DHA, as well as their quantitative relationship, were assessed using radioactive tracers. We hypothesized that a decreased bioavailability of DHA-EE, as compared to free DHA, would result in decreased DHA accretion in key target tissues, including the liver and brain, with potential implications for metabolic effects of treatment.

## Materials and methods

### Animals

The experimental procedures were approved by the Gothenburg ethics review committee on animal experiments (licence #157–2014 and #17–2016) and were in accordance with Swedish laws on the use and treatment of experimental animals. Animals were kept in a facility accredited by Association for Assessment and Accreditation of Laboratory Animal Care (AAALAC) and they were attended daily. Sixteen adult male Wistar rats (Harlan, Netherlands) were maintained in a temperature-controlled (20–22°C) room with a 12 h light–dark cycle (lights on at 06:00) with free access to tap water and a low-fat, carbohydrate-rich, standard rodent chow diet (R70, Lactamin AB, Stockholm), consisting of 4.5% fat, 60% carbohydrate and 14.5% protein by weight (energy content 1254 kJ/100 g). The lipids in the chow are from oatmeal, barley, wheat bran and flour, linoleic acid being the major unsaturated fatty acid.

Seven days before the acute experiment, rats underwent aseptic surgery under anesthesia with isoflurane (Forene®, Abbott Scandinavia AB, Solna, Sweden). After thorough shaving, cleansing with Descutan^®^ (Fresenius Kabi AB, Uppsala, Sweden) and disinfection of the surgical field with chlorohexidine (Fresenius Kabi AB), a polyurethane catheter (C30PU-RJV1303, Instech, Plymouth Meeting, PA, USA) was implanted in the right jugular vein. The catheter was tunneled under the skin up to the neck and connected to a vascular access button (VAB95BS, Instech). The catheter was filled with a “lock” solution (TCS™, Access Technologies, Skokie, Il, USA) through the vascular access button to prevent clotting. Buprenorphine (Temgesic^®^, 1.85 μg/kg, RB Pharmaceuticals Ltd, Berkshire, GB) was given subcutaneously for analgesia. Prophylactic antibiotics were administered *per os* the day before, on the same day and the day after surgery (sulfamethoxazole, 23.3 mg/kg, trimethoprim, 4.4 mg/kg, Bactrim^®^, Roche AB, Stockholm, Sweden).

### Pre-treatment with OM3CA or OM3EE

Animals were adapted to the oral gavage procedure and OM3 formulations, by pre-treatment for 5 consecutive days prior to the acute experiment, with the same formulation (excluding tracer) that they would receive on the acute experiment day. Rats were assigned to either the OM3CA (n = 7) or OM3EE (n = 9) groups and received daily gavages at 10:00 of a fixed volume (240 μL) of the capsule content of either OM3CA (Epanova®) or OM3EE (Omacor®), respectively. To minimize oxidative degradation, formulations were drawn directly into the gavaging syringe from the capsules via a syringe needle immediately prior to each gavage occasion. Nominal daily doses were: OM3CA group; DHA ~0.42 mmol/kg, EPA ~1.27 mmol/kg; and the OM3EE group; DHA-EE 0.73 mmol/kg, EPA-EE 0.98 mmol/kg.

### Tracers and formulations for the acute study

[1-^14^C] Docosahexaenoic acid (^14^C-DHA) and [1-^14^C] Docosahexaenoic ethyl ester (^14^C-DHA-EE) (Quotient Bioresearch Radiochemicals Ltd, Cardiff, GB) with specific activity 2.15 GBq/mmol were assessed a couple of days prior to each experiment and purified (see below) as needed in-house to ensure >97% radiochemical purity.

Tracers were purified by preparative high pressure liquid chromatography (HPLC) on a XBridge C_18_ column (10 μm, 250 mm, ID 19 mm) using a gradient of 30 to 95% acetonitrile in 0.1% H_2_O/trifluoro acetic acid buffer over 15 min, then 95% acetonitrile for 10 min with a flow of 15 mL/min. The compounds were detected by UV light at 234 nm. All solvents used were degassed by purging with helium. The stock solutions of ^14^C-DHA or ^14^C-DHA-EE were concentrated and residues redissolved in dimethyl sulfoxide (DMSO) prior to purification. The fractions containing product were evaporated/co-evaporated with ethanol. The products were dissolved in toluene and concentrations determined by liquid scintillation. Aliquots of the individual tracers containing ~13 MBq (sufficient for 4 rats) of either ^14^C-DHA or ^14^C-DHA-EE were stored in toluene under N_2_ at -70°C until the acute experiment.

Approximately 2 hours before the acute tracer experiments, tracer aliquots were evaporated under N_2_ at room temperature. For the OM3CA group, the content of a capsule (~1 mL) of OM3CA was added to ~13 MBq ^14^C-DHA. For the OM3EE group, the content of a capsule (~1 mL) was added to ~13 MBq ^14^C-DHA-EE. Formulations were thoroughly mixed under a N_2_ stream using a magnetic stirrer for 30 min before dosing. The ^14^C-activity in each tracer infusate was measured. Nominal dosing of DHA and EPA equivalents were as given above (see Pre-treatment with OM3CA or OM3EE). The contribution of the tracers to the total formulation DHA and DHA-EE doses was trivial (1.2 and 0.6% of the nominal doses, respectively) due to the high specific activities of the tracers used (see above) and could be ignored.

### Acute tracer experiment

On the experiment day, rats were fasted for 7 hours before the acute tracer study and were connected to the swivel system at 08:30 and allowed to acclimatize for 1.5 hour. The jugular vein catheter was maintained patent with a continuous infusion of sterile saline solution containing sodium citrate (20.6 mmol/L) at a rate of 10 μL/min. Two basal blood samples (150 μL) were taken 30 and 15 min before dosing. After oral administration by gastric gavage at 10:00, of either OM3CA/^14^C-DHA or OM3EE/^14^C-DHA-EE, blood samples (200 μL) were taken at 10, 15, 20, 30, 45, 60, 120, 180 and 240 minutes after dosing. Blood was collected in EDTA tubes (Microvette® CB 300 K2E, Sarstedt, Nümbrecht, Germany) and immediately centrifuged at 10000 rpm in 4°C for 1 min. An aliquot of 25 μL plasma was added to 2 mL of lipid extraction mixture and the rest of the plasma was transferred to a 0.5 mL micro-tube with cap (72.730, Sarstedt) and frozen for later analysis of triglyceride (TG) and free fatty acid (FFA) levels.

After collection of the last blood sample, rats were given an over-dose of Na-pentobarbital (Allfatal vet. 100 mg/mL, Omnidea, Stockholm, Sweden) and the following tissues were collected for analysis: red quadriceps (RQ), white quadriceps (WQ), liver, cerebellum and epididymal adipose tissue (EAT). Tissue pieces (~100 mg) were weighed and placed in small cellulose cones (Combusto-Cones™, Perkin Elmer) for combustion. Total liver weight was measured.

### Lipid extraction and separation of neutral and polar species

Quantitative lipid extraction was performed based on the method developed by Dole [17]. An aliquot of 25 μL freshly collected plasma, was pipetted directly into glass tubes containing 2 mL 2-propanol: heptane: 1 mol/L acetic acid (40:10:1 vol). Extraction tubes were stored at -20°C for later analysis. To each lipid extraction tube was added 1.2 mL 1 mol/L acetic acid and then 2 mL iso-hexane. Samples were vigorously mixed for 10 min on a multi-tube vortexer after which two phases were allowed to spontaneously completely separate. Tubes were placed in an ethanol/dry ice bath until the lower, aqueous phase was frozen ~10 min. The organic phase was then poured off into new disposable glass tubes. Another 2 extractions were made with 2 mL isohexane added to the thawed aqueous phase in the extraction tubes, according to the same procedure with vortexing, freezing and decanting of the organic phase. Second and third organic phase extractions were added to the initial organic extract.

The organic phase was evaporated to complete dryness at 37–40°C under a stream of N_2_. Just prior to the separation step, lipids were redissolved in 2 mL methyl tert-butyl ether (MTBE) and mixed for ~30 seconds. SPE columns (ISOLUTE®, NH_2_ 200 mg, 3 mL, Biotage AB, Uppsala, Sweden) were activated by adding 2 mL MTBE to each column and eluent was discarded. Lipid extract was transferred to the SPE column and 2 mL of MTBE was added two times to wash neutral lipid species through the column and eluent was collected into scintillation bottles (20 mL LCS Vial, Perkin Elmer, Hägersten, Sweden). To elute polar lipid species, 3 mL 5% acetic acid in MTBE was added to the lipid extract tubes, which were mixed for 30 seconds before transfer to the SPE column. Eluent was collected into a separate set of scintillation bottles and another 3 mL 5% Acetic acid in MTBE was added to the original extraction tubes, without mixing and transferred to the SPE columns and eluent was collected. To each lipid fraction 10 mL of Optiphase Hisafe 3 (Perkin Elmer) was added and mixed awaiting counting.

Control experiments were performed to investigate to what extent phosphatidylcholine is eluted as polar lipid species. Radiolabeled phosphatidylcholine (L-α-DiPalmitoyl-[DiPalmitoyl-1-^14^C]-Phosphatidylcholine (^14^C-PC, NEC682010UC, Perkin-Elmer) was used in this experiment. Ten μL of ^14^C-PC was diluted with 300 μL buffer (phosphate buffered saline, pH 7.4, Gibco®, Life technologies, Paisley, UK) and the solution was put on a magnetic stirrer for 30 minutes. Five replicates with 15 μL of ^14^C-PC solution and 10μL rat plasma added in 2 mL 2-propanol: heptane: 1 mol/L acetic acid underwent lipid extraction as described above. About 80% of radiolabeled PC was recovered in the organic phase and about 2% of radiolabeled PC was recovered as neutral lipid species and less than 0.5% was recovered as polar lipid species.

### Determination of total tissue ^14^C-content

Total tissue ^14^C-activity was determined using a Packard System 387 Automated Sample Preparation Unit (Packard Instrument Co., Inc., Meriden, Connecticut, USA), which completely oxidizes the tissue sample into ^14^CO_2_ which is quantitatively trapped by Carbo-Sorb® (PerkinElmer) in scintillation vials. Following addition of liquid scintillant (Permafluor, PerkinElmer) and mixing, the samples were ready for counting.

### Liquid scintillation counting

The ^14^C-activity in liquid scintillation cocktails from plasma lipid extraction/separation and total tissue oxidation samples were counted using liquid scintillation spectrometry (LS 6500 Multi-Purpose Scintillation Counter, Beckman Coulter™, USA).

### Plasma TG and FFA levels

Enzymatic colorimetric methods were used for quantitative determination of plasma FFA (NEFA-HR(2), Wako Chemicals GmbH, Neuss, Germany) and TG, with glycerol blanking (Trig/GB, ref# 11877771, Roche Diagnostics GmbH, Mannheim, Germany). These analyses were performed on an ABX Pentra 400 (HORIBA ABX Diagnostics, Kyoto, Japan).

### Data analysis and statistics

Areas under the curves (AUCs) of the ^14^C-lipid species, based on discrete time point measurements of the polar and neutral lipid species (AUC_PL_ and AUC_NL_, respectively), were assessed using trapezoidal approximations. Student’s t-tests, comparing group means, were performed using the program GraphPad Prism^®^ 6 for windows (GraphPad Software Inc, San Diego, California, U.S.A.).

In order to examine the contributions of the different forms of circulating DHA (polar vs neutral lipid species) to tissue uptake and retention of DHA the following model (Eq 1) was applied:

$$R=K_{PL}\times AUC_{PL}+K_{NL}\times AUC_{NL}$$ (1)

where the measured variables in this equation are: R, which is the tissue ^14^C-retention at 240 min (dpm/100g) and; AUC_PL_ and AUC_NL_ are the areas under the curves from 0 to 240 min for the polar and neutral lipid forms, respectively, of ^14^C-activity (dpm x min/ml). The unknowns, *K*_*PL*_ and *K*_*NL*_ are the tissue specific clearance rates (ml/100g/min) of the polar and neutral lipid species, respectively. Model fitting and clearance rate estimation were performed using multiple regression analysis (IBM SPSS Statistics Subscription, IBM Corp, USA). Initially, we wanted to investigate whether there was a difference between the OM3CA and OM3EE formulations in the dependence of the tissue retention on the circulating lipids. This would be reflected by differences in the clearance parameters and was addressed by fitting the model (Eq 2):

$$R=K_{PL}\times AUC_{PL}+\Delta K_{PL}\times AUC_{PL}\times G+K_{NL}\times AUC_{NL}+\Delta K_{NL}\times AUC_{NL}\times G$$ (2)

where G = 0 or 1, for the OM3CA and OM3EE data, respectively, and the Δ*K* terms are the differences between OM3EE and OM3CA. p<0.05 was considered statistically significant. Data were expressed as means±SEM.

## Results

One week following surgical implantation of a jugular sampling catheter, both groups had gained weight (~12 g) above the pre-surgical level. Body weights and fasting levels of plasma TG and FFA, immediately prior to oral administration of the fish oil formulations were similar in the OM3CA (n = 9) and OM3EE (n = 7) groups of Wistar rats (Table 1). Total liver weights at the end of the study were for OM3CA, 14.4±0.5 g and for OM3EE, 13.2±0.6 g.

**Table 1:** Body weights and fasting plasma TG and FFA levels immediately prior to oral administration of either OM3CA or OM3EE in male Wistar rats.

	OM3CA	OM3EE
Body weight (g)	336±6	352±14
Plasma TG (mmol/L)	0.74±0.09	0.64±0.07
Plasma FFA (mmol/L)	0.71±0.04	0.82±0.05

Values are means±SEM (OM3CA, n = 9; OM3EE, n = 7).

### Appearance of ^14^C-lipids in plasma following oral administration of either ^14^C-DHA/OM3CA or ^14^C-DHA-EE/OM3EE

**Fig 1:**
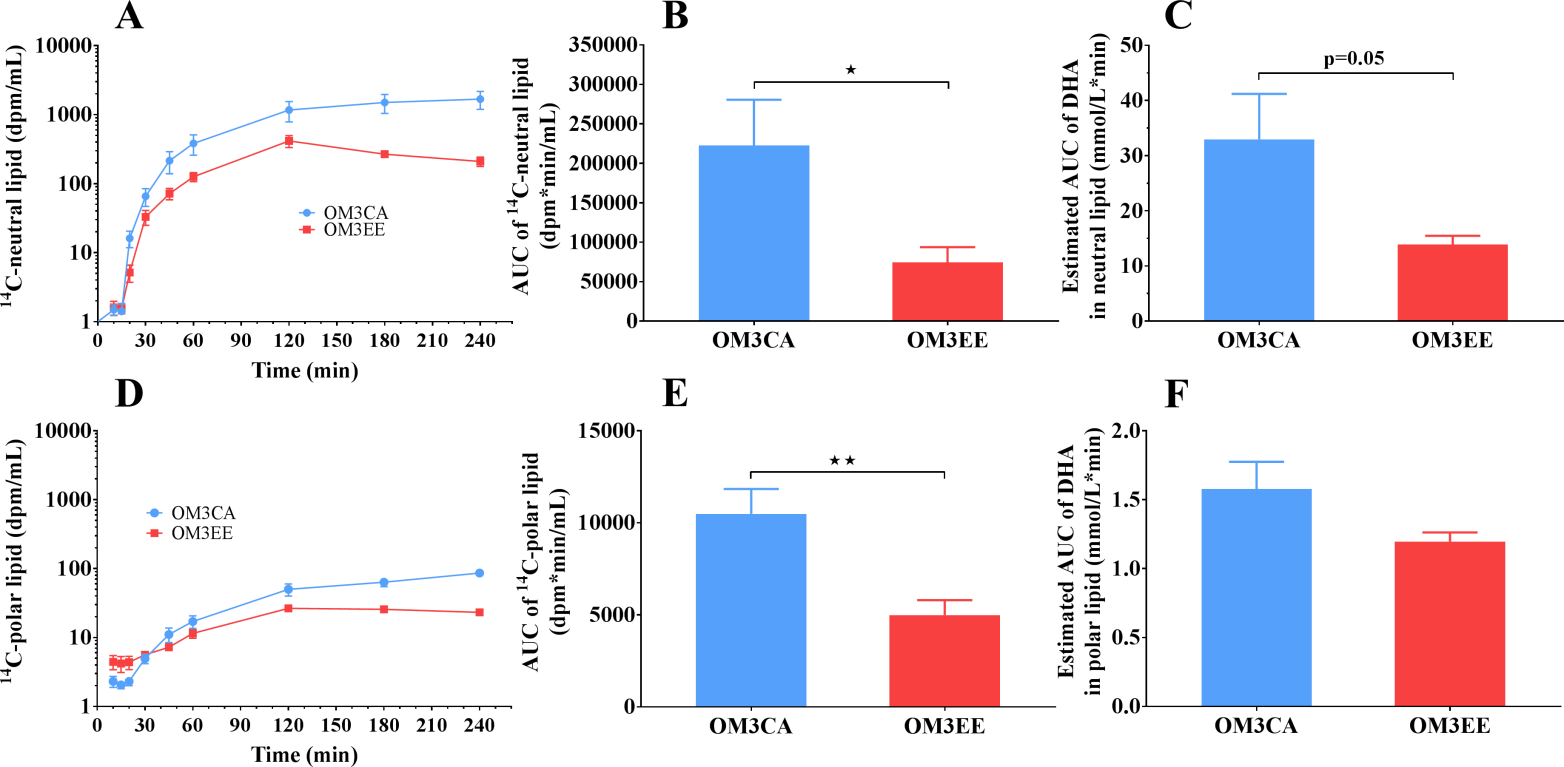
Plasma lipid responses to oral administration of either ^14^C-DHA/ OM3CA or ^14^C-DHA-EE/ OM3EE-EE in Wistar rats. Lipid species were separated into neutral- (A-C) and polar-species (D-F). Time courses for the ^14^C-lipids, in dpm/ml, were normalized to doses of 10^6^ dpm (panels A and D). Areas under the ^14^C-lipid curves, from 0 to 240 min (AUC), are presented in panels B and E. Estimates of the AUCs for DHA, based on the specific activity of the dose formulations, are given in panels C and F. Values are means±SEM (OM3CA, n = 9; OM3EE, n = 7). dpm = disintegrations per minute. *p<0.05, **p<0.01 versus OM3CA, Student’s t-test.

The appearance of lipids in the plasma following oral dosing of either the ^14^C-DHA/OM3CA or the ^14^C-DHA-EE/OM3EE mixtures is shown in Fig 1. Plasma lipids were separated into neutral (Panels A-C) or polar (Panel D-F) species. The appearance of ^14^C-activity in the neutral lipid fraction (Fig 1, panel A), likely esterified DHA in the form of triglyceride, was delayed by about 20 min, probably reflecting transport delay of chylomicrons via the lymphatic system into the blood. The appearance curves were normalized for the ^14^C-tracer dose (10^6^ dpm) enabling direct comparison of the two groups. ^14^C-DHA/OM3CA administration resulted in greater plasma ^14^C-neutral lipid exposures, compared to ^14^C-DHA-EE/OM3EE dosing consistent with higher bioavailability of the OM3CA form of DHA. Thus, the area under the ^14^C-neutral lipid curve, from 0 to 240 min (AUC) was significantly higher with OM3CA compared to OM3EE dosing (Fig 1, panel B, P<0.05, t-test). Correcting the tracer appearance data for the nominal DHA and DHA-EE specific activities of the dose formulations indicates greater oral exposure from the FFA vs the ester form of DHA. Thus, a lower nominal dose of OM3CA (146 μmol/rat versus an equivalent of 256 μmol/rat for OM3EE) tended to result in a higher estimated DHA AUC in the neutral lipid fraction (Fig 1, panel C).

The appearance of ^14^C-activity in the polar lipid fraction in plasma, likely the FFA form of DHA, started to increase above virtually undetectable levels about 30 min after oral dosing i.e. delayed with respect to earlier emergence of the ^14^C-neutral lipid. ^14^C-DHA/OM3CA administration resulted in greater plasma ^14^C-polar lipid exposures, compared to ^14^C-DHA-EE/OM3EE dosing, again consistent with higher bioavailability of the OM3CA versus the OM3EE form. Thus, the area under the ^14^C-polar lipid curve, from 0 to 240 min (AUC) was significantly higher with OM3CA compared to OM3EE dosing (Fig 1, panel E, p<0.01, t-test). Correcting the tracer appearance data for the nominal DHA and DHA-EE specific activities of the dose formulations confirmed the greater oral bioavailability for the free fatty acid versus the ester form of DHA. Thus, a lower nominal dose of OM3CA (see above) did not result in lower DHA AUC in the polar lipid fraction (Fig 1, panel F).

### Uptake and retention of DHA in tissues following oral administration of either ^14^C-DHA/OM3CA or ^14^C-DHA-EE/OM3EE

**Fig 2:**
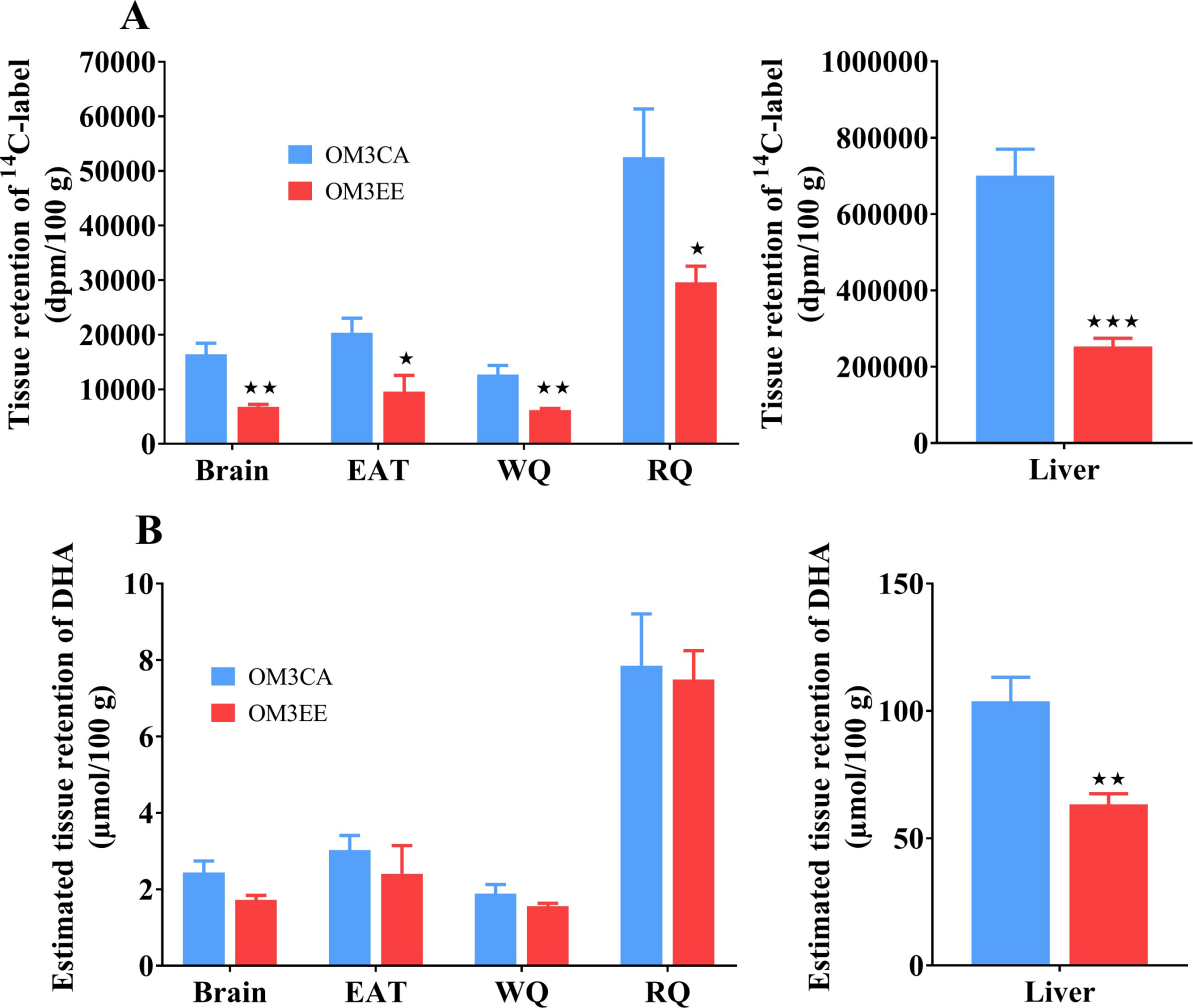
Tissue uptake/retention of DHA, 4 h after oral administration of ^14^C-DHA/ OM3CA or ^14^C-DHA-EE/OM3EE in rats. (A) Tissue retention of ^14^C-label normalized to ^14^C-doses of 10^6^ dpm. (B) Estimated tissue retention of DHA based on the specific activity of the dose formulations. EAT = epididymal adipose tissue. WQ = white quadriceps muscle. RQ = red quadriceps muscle. Values are means±SEM (OM3CA, n = 9; OM3EE, n = 7). dpm = disintegrations per minute. *p<0.05, **p<0.01, ***p<0.001 versus OM3CA, Student’s t-test.

The retention of ^14^C-label, 4 h after oral administration of either ^14^C-DHA/OM3CA or ^14^C-DHA-EE/OM3EE in a number of tissues is shown in Fig 2 (Panel A). Retention of ^14^C-label varied across the different tissues, with the highest uptake in liver, intermediate uptake in red skeletal muscle (RQ) and low uptake in white skeletal muscle (WQ), white adipose tissue (WAT) and the brain. In all tissues examined, the OM3CA formulation resulted in higher tissue incorporation of ^14^C-label than OM3EE dosing. Based on measured total liver weights and the hepatic ^14^C-activity, fractional retention of the dose in the liver at 4 h was estimated to be higher with OM3CA (10.0±1.0%) than with OM3EE (3.2±0.2%) dosing (p<0.001). Estimates of the DHA retention were made based on the tissue ^14^C-data and the specific activities of the dose formulations (Fig 2, panel B). Thus, despite higher dosing of DHA (equivalents) in the OM3EE formulation, the liver DHA retention was significantly lower than that achieved by the OM3CA formulation (Fig 2, panel B). In the other tissues, the lower bioavailability of DHA-EE was compensated for by the higher equivalent dose of DHA resulting in similar estimated DHA retentions for the two formulations.

The dependence of tissue ^14^C-DHA retention on the circulating polar and neutral lipid forms of DHA was examined using Eq 1. An important assumption of this multiple regression analysis is that the independent variables (AUCs) are not correlated. Poor correlation between the AUC_PL_ and AUC_NL_ pairs for individual experiments was confirmed with Pearson’s correlation coefficients of 0.44 (p>0.05) and 0.18 (p>0.05) for the OM3CA and OM3EE groups, respectively and a value of 0.30 (P>0.05) for the pooled data set, excluding one outlier.

Based on application of Eq 2, we found no evidence in any tissue examined for a difference in *K* values between the OM3EE and OM3CA groups. Specifically, the Δ*K* terms in Eq 2 were not found to be significantly different from 0. Thus, there was no evidence for any difference between OM3EE and OM3CA in the relationship between the tissue retention of DHA and the plasma exposures of the two forms of DHA. This allowed us to ignore treatment group and analyze the pooled data set using Eq 1.

**Fig 3:**
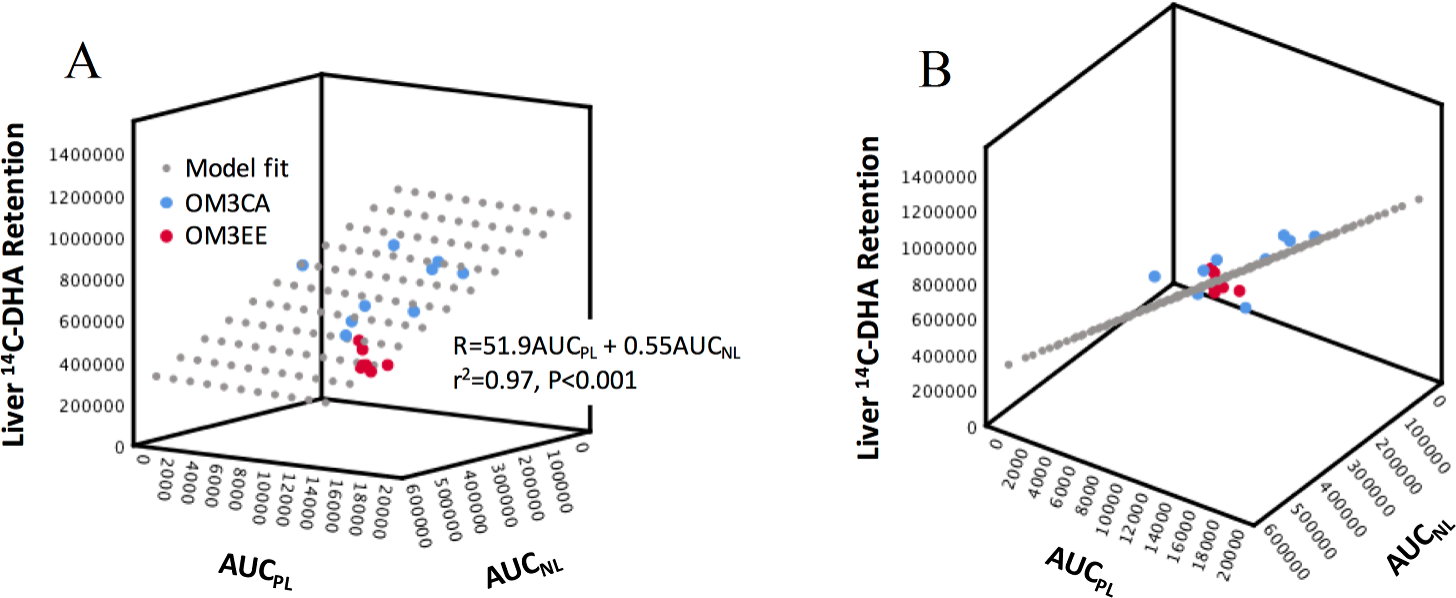
Dependence of liver uptake/retention of DHA (R) on plasma exposure of DHA in either polar lipid (AUC_PL_) or neutral lipid (AUC_NL_) forms. (A) A plot of the best fit plane given by the equation for R and represented by the grey dots projecting out of the inner corner of the diagram, as well as, the data points for the OM3CA (blue dots) and OM3EE (red dots) groups. (B) Shows the same 3D data set as in A, following a rotation of the vertical axis to view the (model fit) plane, edge on, revealing the data point residuals. Units: Liver ^14^C-DHA Retention at t = 240 min, dpm/100g; AUC_PL_, dpm.min/ml; AUC_NL_, dpm.min/ml.

Eq 1 provided a good description of the relationship between the ^14^C-retention data and plasma exposures, with r^2^ > 0.84 and significance values for the overall model fit F-statistic of p<0.001 for all tissues examined. Table 2 summarizes the best fit *K*_*PL*_ and *K*_*NL*_ estimates. In all tissues examined, uptake/retention of DHA was apparently most strongly determined by the circulating polar lipid. Only in the liver, was there a significant additional component coming from the neutral lipid. Even in this tissue, however, the contribution from polar lipid (*K*_*PL*_ × AUC_PL_ /R) was estimated to be 85±2% (range 65 to 95%) with only 15±2% (range 5 to 35%) from neutral lipid. A 3D visualization of the liver data versus AUC_PL_ and AUC_NL_ and linear model fit (which describes a plane) is shown in Fig 3. Fig 3 (Panel A) shows the greater dependence of liver retention of DHA on circulating polar versus neutral lipid: by comparing the slopes of the model fit plane intersection with the Retention×AUC_PL_ and Retention×AUC_NL_ backplanes. Fig 3 (Panel B), oriented to illustrate data-fit residuals, confirms that the model provides a description of both OM3CA and OM3EE group data. In all other tissues, according to the linear model, there was no significant, direct contribution of the circulating neutral lipid to the uptake/retention of DHA with *K*_*NL*_ indistinguishable from zero.

**Table 2:** Dependence of tissue uptake/retention of DHA on plasma exposure of DHA in either polar lipid or neutral forms.

Tissue	*K*_*PL*_(mL/100g/min)	*K*_*NL*_(mL/100g/min)
Liver	51.9±5.1***	0.55±0.21*
Brain	1.40±0.20***	0.007±0.008
EAT	1.58±0.38**	0.014±0.016
RQ	4.40±1.03**	0.028±0.044
WQ	1.11±0.18***	0.003±0.007

EAT, epididymal white adipose tissue; RQ, red quadriceps; WQ, white quadriceps. Results show best fit parameter estimates ± SEM.

*p<0.05,

**p<0.001,

***p<0.001 is the probability that the *K*_*PL*_ or *K*_*NL*_ estimates significantly differ from zero. Multiple linear regression analysis was used to obtain estimates of tissue-specific clearance rates of either the polar lipid form of DHA (*K*_*PL*_) or the neutral lipid form (*K*_*NL*_) from the relationship between retained DHA in the tissue and plasma AUCs for polar and neutral DHA, respectively. Estimates were obtained from pooled results of both OM3CA and OM3EE groups (n = 16).

## Discussion

We compared the plasma appearance and tissue accretion of docosahexaenoic acid (DHA) in response to oral dosing of free carboxylic acids and ethyl ester forms of this omega-3 fatty acid. The results are consistent with markedly higher bioavailability of the free carboxylic acids compared to ethyl ester forms, culminating in an increased accretion of DHA in the liver. Our analysis suggests that following entry into the plasma, the transport, tissue distribution and metabolic fate of DHA is independent of the dosing form. Thus, we found no evidence for a change in tissue distribution associated with reduced bioavailability. In plasma, DHA is transported as both polar and neutral lipid forms. Evidence is provided that polar lipid is, by far, the quantitatively most important form, driving uptake into a range of tissues (skeletal muscle, fat and brain). Only to the liver was there an additional, smaller, but significant contribution from neutral lipid in plasma.

Results from this study are consistent with a number of previous studies in animals and humans showing greater systemic bioavailability of free omega-3 fatty acids compared to omega-3 fatty acid ethyl esters [6–14]. Furthermore, the current work verifies that, compared to the ethyl ester form of DHA, dosing of the free carboxylic acids has a higher efficiency of DHA accretion across the observed range of tissue types: liver, skeletal muscle, white adipose tissue and brain. Indeed, we calculated that despite the nominally lower dose of DHA equivalents given, the OM3CA formulation actually achieved significantly higher absolute levels of DHA deposition in the liver. This is of potential importance to efficacy, as a major tissue locus of the therapeutic effect of omega-3 fatty acid treatment on plasma lipids is thought to be the liver [18]. Published evidence indicates that the difference in OM3CA and OM3EE bioavailability is especially pronounced in situations where pancreatic lipase is limited [9, 10], probably because absorption of the active moiety of OM3EE, requires cleavage of the ethyl ester by the action of this enzyme in the intestinal lumen [11]. The fasted, low-fat diet fed rat used in the current study is a model, which also has a relative limitation in pancreatic lipase. Thus, increasing dietary fat content above the level in the currently used model (12% of calories) has been shown to increase pancreatic lipase in the rat [19]. Thus, a higher fat intake, mimicking the human situation, would have reduced the difference in systemic bioavailability between the two formulations.

Our analysis of the relationship between tissue accretion and systemic exposure of DHA suggests that the quantitatively most important direct route of DHA delivery to the tissues is in the form of a polar, rather than neutral, lipid. Thus, accretion of labelled DHA in skeletal muscle, adipose tissue and brain is approximately proportional to exposure of labelled polar lipid and (linearly) independent of the neutral lipids. Based on previous studies [20, 21], the dominant polar lipid form is probably free carboxylic acids (i.e. FFA), whereas, the dominant neutral lipid form is esterified DHA in TG [22]. To estimate the contribution of phospholipids to the polar fraction, we did a control experiment using radiolabelled phosphatidylcholine. The results indicate that a minor fraction of phosphatidylcholine and therefore likely also other phospholipids contribute to the polar lipid species. It is therefore likely that uptake of DHA into the above tissues is driven by FFA rather than TG. The partial regression coefficients, *K*_*PL*_ and *K*_*NL*_, obtained from the multiple linear regression analysis could in fact be equivalent to plasma clearance rates into the tissues of polar and neutral lipid species, respectively. For this to be true, a major requirement is that the tissue pool behaves as a sink, i.e. that all DHA entering this pool over the experimental period from the plasma is retained, as discussed in [23]. This assumption is probably a reasonable approximation given that the majority of the tissue DHA tracer is likely in the form of structural and storage lipid [24]. The clearance rate estimates, which index the ability of the tissues to take up and store DHA, varied in a tissue-specific manner with by far the highest value in liver, followed by red skeletal muscle and lower levels in fat, brain and white skeletal muscle. Interestingly, a similar pattern of tissue specific clearance rates is seen for the long chain FFA, palmitate [25, 26]. Our results in the brain agree well with a detailed kinetic study, which concluded that the plasma free carboxylic acid form was the major pool supplying this tissue with a negligible contribution from plasma DHA esterified into TG [20]. Remarkably, even at a quantitative level, results from the two studies are very similar, with Chen *et al* reporting a brain clearance rate of plasma DHA, as carboxylic acid, into storage products of ~1.2 mL/100 g/min versus our estimate of 1.4 mL/100 g/min. Only in the liver was there a significant additional contribution from labelled neutral lipid. This neutral lipid most likely represents flux of TG associated with hepatic clearance of chylomicron remnants. Finally, there is no evidence for a difference between the DHA circulating lipid forms for orally dosed free carboxylic as compared to ethyl ester forms, which is perhaps not that surprising given that, in both cases, it is the free carboxylic acid form of DHA that is absorbed.

What could be the source of the polar lipid form of DHA driving uptake into tissues? Orally administered DHA enters the circulation, as other longer chain fatty acids, esterified to TG, phospholipids and to a lesser extent cholesterol esters and transported as chylomicrons. DHA esterified in TG and phospholipids are then released as free DHA, through the action of lipoprotein lipase (LPL) in the tissues. It is known for long-chain chain fatty acids that some of the liberated fatty acid is not taken up locally, but instead, escapes into the systemic circulating FFA pool [27]. Indeed, this transport route may be especially exaggerated for long chain omega-3 fatty acids. Lucinda *et al* [28] provided evidence for a substantial escape of both DHA and EPA, liberated by LPL, from the adipose tissue capillaries into the plasma FFA pool. In response to a meal containing a mixture of fatty acid species, including these omega-3 fatty acids and palmitate, adipose tissue release of the free omega-3 fatty acids increased markedly, while palmitate release was suppressed. This also fits well with the observationthat only very small quantities of DHA and EPA are stored in the adipose tissue, suggesting a limited storage ability in this tissue [29].

There are several limitations of the current study. Firstly, we have not specifically identified the circulating labelled species but rather, for simplicity and robustness, broadly classified them based on the presence or absence of charge using a lipid separation procedure. We assume, in the discussion above, that the polar lipid fraction is predominantly free carboxylic acids. However, in addition plasma DHA also exists as polar lysophosphatidylcholine esters [20, 21], which depending on the relative concentration and tissue clearance rates could influence the interpretation of our clearance rate estimates. It is worth pointing out that although in vivo retroconversion of DHA to EPA may have occurred in our study, it probably did not influence the reported tracer based parameters since this requires a cycle of β-oxidation [30], which would result in exclusion of the radiolabel from the lipid fractions. A second limitation is that we cannot conclude to what extent the FFA in the polar fraction are derived from hydrolysis of TG as compared to phospholipids. Also, we have not measured radioactivity in the different esterified lipid classes and therefore the contribution of DHA in TG as compared to phospholipids for the uptake of whole particles in the liver is unclear. A third limitation concerns the fact that our results only provide information about DHA transport and tissue distribution for a relatively short duration after oral dosing. Another potential limitation of the study is that the EPA to DHA ratios are different in the OM3CA and OM3EE formulations. A differential affinity for a common transporter or enzyme step involved in the metabolism of EPA and DHA in combination with saturable kinetics could result in a lack of correspondence between the relative dose and relative plasma exposure. Therefore, we cannot exclude that there is an interaction between the two fatty acids that would influence the kinetics of DHA. The tissue distribution at 4 hr is unlikely to accurately represent the fate of DHA at later time points. In particular, the liver takes up a large fraction of the plasma DHA, but the liver pool turns over relatively rapidly, subsequently releasing the initially accreted DHA in VLDL over a period of hours [31]. At the same time brain levels progressively increase, perhaps more dependent on supply of plasma lysophosphatidylcholine esters compared with the free DHA over the longer term [21].

In conclusion, our study based on clinically approved omega-3 formulations for the treatment of severe hypertriglyceridemia, confirms the higher oral bioavailability, both at the systemic and target tissue levels, of the free carboxylic acid versus ethyl ester form of DHA, in a preclinical model of limited pancreatic lipase activity. In addition, we provide evidence based on a quantitative analysis that following absorption the plasma transport and tissue distribution of DHA is independent of these dosed forms. Furthermore, plasma free carboxylic acids, rather than TG esters likely supply the major flux of DHA to the tissues.
